# Clinicians' Perspectives on Negative Effects of Psychological Treatments

**DOI:** 10.1080/16506073.2014.939593

**Published:** 2014-09-09

**Authors:** Samuel Bystedt, Alexander Rozental, Gerhard Andersson, Johanna Boettcher, Per Carlbring

**Affiliations:** ^a^ Division of Clinical Psychology, Department of Psychology, Stockholm University, Stockholm, Sweden; ^b^ Department of Behavioural Sciences and Learning, Linköping University, Linköping, Sweden; ^c^ Swedish Institute for Disability Research, Linköping University, Linköping, Sweden; ^d^ Division of Psychiatry, Department of Clinical Neuroscience, Karolinska Institutet, Stockholm, Sweden; ^e^ Department of Clinical Psychology and Psychotherapy, Freie Universität Berlin, Germany

**Keywords:** negative effects, adverse events, Cognitive Behavior Therapy, online survey, thematic analysis

## Abstract

Negative effects of psychological treatments is a fairly unexplored area of clinical research. Previous investigations have indicated that a portion of all patients experience negative effects in terms of deterioration and various adverse events. Meanwhile, evidence suggests that many clinicians are untrained in identifying negative effects and unaware of the current research findings. The objective of the current study is thus to investigate clinicians' own perspectives and experiences of possible negative effects of psychological treatments. An invitation to participate in an anonymous online survey consisting of 14 open-ended questions was distributed via three mailing lists used by clinicians that primarily identify themselves as cognitive behavior therapists. The responses were analyzed using a qualitative method based on thematic analysis. In total, 74 participants completed the survey. A majority agreed that negative effects of psychological treatments exist and pose a problem, and many reported having experienced both deterioration and adverse events among patients in their own practice. The thematic analysis resulted in three core themes: characteristics of negative effects, causal factors, as well as methods and criteria for evaluating negative effects. The clinicians recognize that negative effects exist, but many are unaware of the current research findings and are unfamiliar with methods and criteria for identifying and preventing deterioration and adverse events. The results provide evidence for further dissemination of the present knowledge regarding negative effects, particularly during basic clinical training, as well as the need for raising awareness of the available methods for identifying and preventing negative effects.

## Introduction

Psychological treatments have primarily been evaluated with regard to their potential for preventing and alleviating mental distress (Boisvert, 2010). Meanwhile, little is known about the occurrence and characteristics of possible negative effects, reflecting a major shortcoming in clinical research (Nutt & Sharpe, 2008). Investigations of negative effects have almost exclusively focused on the so-called fringe psychotherapies, e.g., rebirthing and recovered memory techniques, while paying less attention to negative effects that might be associated with evidence-based care (Barlow, 2010). However, recent findings suggest that many patients experience deterioration and adverse events despite receiving psychological treatments that have been validated and are properly performed (Berk & Parker, 2009). Foulkes (2010) states that any intervention with the potency of relieving mental distress also carries with it the risk of eliciting negative effects. Similarly, Castonguay, Boswell, Constantino, Goldfried, & Hill (2010) argue that clinicians should acknowledge the probability of inadvertently having induced negative effects to one or more patients during the course of psychological treatment, and that clinicians as well as researchers should become more aware of how to monitor and manage situations that may have a negative impact on the therapeutic process and treatment outcome.

To what extent negative effects exist and pose a problem in psychological treatments is, however, a topic of great debate with a long history in clinical research (Boisvert, 2010; Rozental et al., 2014). The first empirical evidence of negative effects is often assumed to be the Cambridge-Somerville delinquency prevention study (Powers & Witmer, 1951), although it was not until a review of a number of outcome studies by Bergin (1966) that sparked the interest of whether psychological treatments can produce negative effects. Referring to it as the deterioration effect, Bergin (1996) proposed that apart from the patients that benefit from psychological treatments, there are also those who do not profit at all, and a small proportion who become worse. Albeit criticized by May (1971) and Rachman (1973) on account of the difficulty to prove a cause-effect relationship between psychological treatments and deterioration, several recent outcome studies have provided evidence that between 5% and 10% of all patients receiving psychological treatments deteriorate (Hannan et al., 2005; Hatfield, McCullough, Frantz, & Krieger, 2010; Heins et al., 2010; Lambert et al., 2002). However, deterioration might not be the only type of negative effect that exist (Boisvert & Faust, 2002). Strupp and Hadley (1976) investigated how a number of influential clinicians and researchers perceived negative effects, and presented a tripartite model in which negative effects should be assessed from the perspective of patient, the clinician, and society, suggesting that mental distress and personal well-being largely depend on the eye of the beholder (Strupp, Hadley, & Gomes-Schwartz, 1977). Instead of using the definition deterioration effect, Strupp and Hadley (1976) put forward the idea of negative effects, assuming that it comprises more than just deterioration, e.g. novel symptoms, misuse of psychological treatments and undertaking unrealistic tasks and goals. A similar concept was proposed by Mays and Franks (1980) using the term negative outcome:

A Negative Outcome is a significant decline in one or more areas of a patient's functioning, between the onset of psychotherapy and termination of therapy (and for controls, over an equivalent period of time), which persists for a substantial period of time beyond termination of therapy. The term Negative Outcome is not restricted to those negative changes which are therapy-induced, and usage of the term does not therefore imply that the therapist is necessarily responsible for the negative change.

Negative outcome thus included both deterioration as well as other types of negative effects, but did not presume that the negative effects were necessarily related to psychological treatments per se (Mays & Franks, 1985). It is for instance plausible that some patients may experience negative effects because of events that occur in their everyday life, and that some patients might actually have deteriorated to a greater degree had they not received any help at all (Mays & Franks, 1980). The natural fluctuation of mental distress also obfuscates the idea of negative effects, making it difficult to distinguish what is caused by psychological treatments and what is part of an ongoing psychiatric condition (Rachman & Wilson, 1980). Furthermore, nonresponse, drop-out, interpersonal difficulties, dependency, and social stigmatization have all been recognized as negative effects that might interfere or prevent the patient to benefit from psychological treatments (Crown, 1983; Foa & Emmelkamp, 1983; Dimidjian & Hollon, 2010). Determining what constitute negative effects is therefore complicated, but a number of suggestions on how to monitor and report negative effects have lately been presented (Peterson, Roache, Raj, & Young-McCaughan, 2013). Linden (2013) provided a comprehensive checklist that divide negative effects into a number of different categories, e.g., events and reactions unrelated to the interventions in use, nonresponse, and deterioration of illness. In addition, Parker, Fletcher, Berk, and Paterson (2013) have developed a questionnaire intended to probe for negative effects among patients undergoing psychological treatments. Nestoriuc and Rief (2012) have also put forward an inventory for assessing negative changes in various life domains, and Dimidjian and Hollon (2010) have suggested that clinicians and researchers should implement both quantitative and qualitative methods to examine the occurrence and characteristics of negative effects. It is therefore reasonably to assume that the investigation of negative effects will become more common in clinical research, and that it can help prevent patients from experiencing deterioration and adverse events despite receiving evidence-based care (Barlow, 2010).

Despite the recent interest in negative effects of psychological treatments among researchers, clinicians might not be as aware and up to date regarding the debate on how to monitor and report negative effects (Castonguay et al., 2010). Investigations of how clinicians perceive and experience negative effects in their clinical practice are scarce, but there are some indications that they may not acknowledge that some patients fare worse and encounter adverse events (Boisvert & Faust, 2003). Strupp and Hadley (1976) distributed a survey on negative effects to 150 researchers and clinicians, of whom 70 responded, with a majority agreeing that negative effects constitute a significant problem in psychological treatments. However, the participants were selected based on their expertise and extensive knowledge in clinical research, making it difficult to extend the results to clinicians in general. Similarly, Boisvert and Faust (2003) explored the understanding of psychotherapy research findings, including the occurrence of negative effects, among 25 prominent researchers, of whom 12 responded and confirmed that about 10% of all patients deteriorate during psychological treatments. The only examination to date of ordinary clinicians comprehension of negative effects is, however, a survey by Boisvert and Faust (2006) that replicated their previous investigation of psychotherapy research findings, but involved 500 randomly selected clinical psychologists of the American Psychological Association. The findings indicated that of the 181 participants whom responded, 28% were unaware that a considerable portion of all patients seems to experience negative effects. This can be seen as particularly alarming when considering the results of Hatfield et al. (2010), exploring 14 clinicians' ability to detect whether one of their patients were about to deteriorate using only their clinical judgement. In only 21% of the cases were the participants able to recognize when a patient had deteriorated, while less than 9% of the cases were detected in terms of nonresponse. Hatfield et al. (2010) found similar evidence when using a survey that was administered to 300 randomly selected clinical psychologist of the American Psychological Association, of whom 36 responded, implying that clinical judgment alone does not seem to be sufficient in order to apprehend negative effects in psychological treatments. Hatfield et al. (2010) concluded that clinicians seem to be overly optimistic with respect to their own performance, lending support for a systematic evaluation of treatment progress, e.g. the Treatment Outcome Package (TOP; Kraus, Seligman, & Jordan, 2005) and the Outcome Questionnaire-45 (OQ-45; Lambert et al., 2003).

The purpose of the current study was to extend the previous investigations of how clinicians perceive and experience negative effects in their clinical practice, as well as to examine their knowledge of the available research findings regarding negative effects in psychological treatments. The current study also seeks to explore whether clinicians were informed about negative effects in their basic clinical training, by what criteria they would judge a patient as having been exposed to negative effects, which factors might be considered responsible for negative effects, and whether there exist certain patient groups at risk of deterioration or adverse events. The overall aim of the current study was thus to provide valuable knowledge of how clinicians regard negative effects of psychological treatments, as well as to further the understanding of how clinicians themselves apprehend and deal with negative effects in their everyday clinical practice.

## Methods

### Procedure

An invitation to participate in an online survey concerning negative effects of psychological treatments was sent out to a select group of individuals in order to conduct a pilot test. After minor revisions regarding the wording of the questions, the survey was distributed via email to the Swedish Society for Behavior Therapy (consisting of approximately 1000 members), the Section for Cognitive Behavior Therapy within the Swedish Psychological Association (consisting of approximately 350 members), as well as students attending the psychotherapist program with a cognitive behavioral orientation at the Department of Psychology at Stockholm University (consisting of approximately 50 students). The survey consisted of 14 open-ended questions (see Appendix), of which 3 were adapted from an earlier investigation of negative effects of psychological treatment among practicing clinicians and researchers (Hadley & Strupp, 1976; Strupp, Hadley, & Gomes-Schwartz, 1977). Participants were guaranteed anonymity and that no meta-data, i.e., IP-addresses, would be logged by the website hosting the online survey or available to the authors of the current study. No financial compensation was offered to those who chose to participate in the survey, except for the students attending the psychotherapist program who were able to receive a book on anxiety upon completion. The survey was accessible for two weeks, during which additional reminders were sent out to the participants. Information with regard to the purpose of the current study, the principal investigators, as well as the possibility to receive a final copy of the results, was included in the invitation.

### Participants

In total, 74 clinicians participated in the survey, of which 45 (60.8%) were women and 28 (37.8%) were men, while one (1.4%) individual choose not to disclose his/her gender. The mean age of the participants was 45.52 years (SD = 10.36), ranging from 26 to 67 years. The mean number of years working as a clinician was 11.98 years (SD = 8.71), ranging from 1 to 39 years. In terms of therapeutic orientation, 63 (85%) of the participants described themselves as Cognitive Behavior Therapists (CBT), 6 (8%) as CBT with a strong influence from Acceptance and Commitment Therapy (ACT), 10 (13.5%) portrayed themselves as Behavior Therapists (BT), and 3 (4%) stated that they were BT using ACT. In addition, one (1.4%) participant identified herself as a Cognitive Therapist, and one (1.4%) participant reported using integrative methods combining BT and theories of attachment.

In Sweden, the title psychotherapist is a licence regulated by the National Board of Health and Welfare (Socialstyrelsen), and can only be obtained by documenting an adequate university degree and sufficient clinical training. Essentially, there are two ways of becoming a psychotherapist: either the individual is a licenced psychologist and completes a three-year education and clinical training within the psychotherapist program, or the individual has a different background in health care or social work that includes basic clinical training which allows application to the psychotherapist program, e.g., licenced nurse, licenced physiotherapist, licenced psychiatrist, licenced social worker, or clerical worker. Hence, it is not uncommon in Sweden to have two separate licences, i.e., licenced psychologist and licenced psychotherapist. A full description of the participants' occupation is illustrated in Table 1.

**Table 1:** Description of the participants' current occupation

Occupation	
Licencsed psychologist	38 (51.4)
Licencsed psychologist and licencsed psychotherapist	23 (31)
Licencsed social worker	5 (6.7)
Licencsed social worker and licencsed psychotherapist	3 (4)
Licencsed psychotherapist (basic clinical training unspecified)	2 (2.7)
Licencsed physiotherapist	1 (1.4)
Licencsed psychiatrist and licencsed psychotherapist	1 (1.4)
Basic clinical training (occupation unspecified)	1 (1.4)

### Analysis

The data-set from the survey consisted of 17.406 words that were explored using thematic analysis. Because the responses were imputed directly by the participant, neither transcription or further processing was warranted in order to proceed with the investigation of the material. A qualitative method based on thematic analysis was used because of its ability to examine a specific concept through the unique perspective of the participants (Braun & Clarke, 2006). Thematic analysis is particularly useful as an inductive approach, identifying recurrent themes that are rooted in the data without having to fit it into a pre existing theoretical framework. This allows the researcher to examine the participants' own comprehension of a given concept, in this case, negative effects of psychological treatments. Thematic analysis has, for instance, previously been employed in relation to patients' experiences of Internet-based Cognitive Behavior Therapy for Bulimia Nervosa (McClay, Waters, McHale, Schmidt, & Williams, 2013), as well as adolescents' perception of diagnostic evaluation in psychotherapy (Binder, Moltu, Sagen, Hummelsund, & Holgersen, 2013), and is a common qualitative method used for analyzing large data-sets in medical and social sciences (Willig, 2013).

The thematic analysis was conducted by the main author of the current study in accordance with the steps proposed by Braun and Clarke (2006): (1) the data were read and reread to get an overview of the material and its content; (2) each response to the open-ended questions was coded using its semantic content, i.e., the meaning of the actual words written, so that all responses carrying similar content would be identified within the data-set. All codes were then named after the semantic content in the responses, for instance, a response concerning the therapeutic alliance received the code “alliance”. Responses that consisted of several semantic contents were given multiple codes and (3) codes were structured into groups with their respective headlines, each describing the meaning of each group. Each code was also given an identification number representing the specific participant, 1–74, and a letter representing the response to a question, *a*–n; (4) themes were generated in an iterative process using both the data-set and the groups of codes, that is, each group was tested against the actual content of the data-set a number of times by returning to the data and rereading and reformulating the themes; (5) the themes were congregated into core themes and, where applicable, sub-themes. Formulation of the final themes in the current study was performed using the semantic meaning of the actual words in the data-set; (6) the results from the thematic analysis was used to investigate the material, relate it to prior research, and discuss the main theoretical findings. In the event of methodological issues or difficulties related to the analytic procedure or investigation of the results, the co-authors were consulted. Furthermore, the themes and formulations of themes were reviewed by the second author in order to dissolve any disagreement and increase reliability.

In addition to the thematic analysis, the responses to the questions 1–6, as well as 11–13, were used to provide descriptive statistics, while the responses to questions 5 and 11 were used both in the thematic analysis and as descriptive statistics. Question 12 only allowed a dichotomous yes or no response to whether or not the participants had received information concerning negative effects during their basic clinical training.

## Results

### Descriptive statistics

In terms of whether the participants believed that negative effects of psychological treatment exist and pose a problem, 73 of 74 participants responded, with 63 participants (94.5%) stating that they agreed. The four (5.5%) participants who disagreed had between 13 and 30 years of clinical practice, and did not differ from the rest of the participants in any significant way. Overall, 55 (75%) participants described that they had clinical experience of negative effects, and eight (11%) responded to having received information about negative effects during their basic clinical training.

### Thematic analysis

The thematic analysis of the data-set revealed 3 distinct core themes and 12 sub-themes, which are illustrated in Table 2: characteristics of negative effects, causal factors, as well as methods and criteria for evaluating negative effects.

**Table 2:** Themes and sub-themes in the data-set

Themes	Sub-themes
Characteristics of negative effects	*Short-term negative effects*
	*No treatment effect*
	*Deterioration*
	*Dependency*
	*Impact on other life domains*
Causal factors	*Incompetence and inadequately applied methods*
	*Potentially harmful treatments*
	*Insufficient therapeutic alliance*
	*Failed ethical judgment and professional conduct*
	*Discontinuing treatment*
	*External factors*
	*Patients at higher risk of suffering from negative effects*
Methods and criteria for evaluating negative effects	*None*

### Characteristics of negative effects

#### Short-term negative effects

Many of the participants described how psychological treatment has the potential of evoking strong but temporary feelings of discomfort. For example, a patient performing exposure in vivo will most likely experience an increase in anxiety, even though it is presumed to be beneficial in the long run. A short-term increase in discomfort was referred to by the participants as an unavoidable part of many interventions, but patients are not always be prepared for this and might choose to end treatment prematurely, which in turn could make them less inclined to seek help for their difficulties in the future. One participant wrote the following:

The patient does absolutely experience aversive events because of treatment, in the short run. If they have the strength and patience to bear with it, they will be empowered by this. However, if they are not prepared, the aversive effects can prove to be long lasting. (Licenced social worker, 47 years old)

#### No treatment effect

Entering treatment and not experiencing any positive results were perceived by some of the participants as a negative effect, particularly because it can generate feelings of hopelessness and lead to the perception of oneself as being damaged beyond repair. One participant described this process as attributing the lack of treatment effect to personal deficiencies:

Yes, for example, if there is no treatment effects, the patient, depending on how one interprets the lack of results, might gain a stronger belief in its own negative self-image (it's my own fault that therapy did not work) or a negative experience of the possibility to receive help from others (there is no help to get, others can't help me). (Licenced psychologist, 44 years old)

Another participant described the experiences of meeting patients with a history of failed treatments and the feelings of hopelessness that sometimes follow:

For me, it's not unusual to see patients, who describe previously failed treatments (this might not qualify as negative effects – but one can view the lack of a treatment effect as something negative, if this represents a lost opportunity to participate in a different and more effective treatment). In my view, lack of treatment effects seems to add to an already existing sense of hopelessness in patients. (Licenced psychologist and licenced psychotherapist, 38 years old)

#### Deterioration

Several participants mentioned deterioration of illness as a negative effect, describing how some patients might fare worse from entering treatment than they would have without it. In addition, several participants also regarded the emergence of new symptoms, e.g., insomnia, low self-esteem, and the manifestation of a sick role, as other examples of negative effects. One of the participants wrote about reinforcement of behaviors that are not in the best interest of the patient:

Escalating suicidal tendencies (within the therapeutic contact) different kinds of reinforcing consequences contingent upon self-harm. Then there are of course a lot of examples of how treatment professionals, motivated by good intentions, have been encouraging avoidance of anxiety at any costs, etc. (Licenced psychologist, 32 years old)

#### Dependency

A few participants illustrated how patients sometimes become dependent on the therapist or on therapy in general, and that therapists often fail to see the treatment as a means of increasing the patient's ability of becoming self-reliant. Particularly, long-term treatments were referred to by the participants as a great risk factor for developing dependency. One participant wrote explicitly about how some patients gain positive effects and at the same time they lose their independence as a result of undergoing treatment:

Sometimes patients can get less independent as a result of participating in treatment. In other words, they can't do things on their own, that ending therapy would be negative because then the problems would return. (Licenced psychologist, 40 years old)

#### Impact on other life domains

The impact of treatment on other domains of life was portrayed by some of the participants as a potential negative effect, particularly with reference to the loss of time for social activities or work. A number of participants mentioned that treatment might require the patient to be on sick-leave from work in order to manage an extensive treatment, e.g., exposure with response prevention for severe obsessive compulsive disorder. One participant wrote about how the path to recovery sometimes is both difficult and invasive:

During more intense and time-consuming treatments such as intensive exposure treatment of OCD, patients might be forced to cut down on other activities, and sometimes they might even need to take sick leave to reserve time for the treatment, which potentially can have aversive effects in other areas of life. (Licenced psychologist, 27 years old)

### Causal factors

#### Incompetence and inadequately applied methods

Many participants in the study perceived incompetence or inadequately applied methods as potential causes of negative effects, highlighting the need for sufficient clinical practice and opportunities for further training among therapists. Several of the participants emphasized the responsibility of the clinician to ensure that skills and knowledge are up to date, as well as the need to acknowledge one's own limitations. A therapist should be aware which patients to treat and which to refer to others. One participant wrote the following about therapist behavior and the necessity to obtain as much information as possible before the treatment is initiated:

Different therapist behaviors: lack of responsiveness, lack of competence by applying treatment methods, lack of time, lacking maintenance program and follow-up. Not checking up on the patient regarding for example homework. But also that the client did not provide all the necessary background information that might have an impact on treatment outcome (e.g. information on previous episodes of depression). (Licenced psychologist and licenced psychotherapist, 51 years old)

Another participant wrote about identifying important markers of treatment failure, that is, the lack of behavior change, and the fact that both the therapist and the patient may reinforce each others' avoidance behaviors:

The biggest problem I see is if there is no change in the patient's behavior as a result of the therapy, or if there is a gradual increase in avoidance behavior, which you are unable to reverse. The patient might feel comfortable to start treatment and simply talk and experience a sense of relief after each session, and at the same time reinforces the therapist's sense of importance. The problem is that this reinforces the behavior of going to therapy to talk, which becomes yet another avoidance behavior. (Licenced psychologist, 59 years old)

#### Potentially harmful treatments

A few participants described potentially harmful treatments as responsible for negative effects, similar to those referred to by Lilienfeld (2007), e.g., rebirthing, grief counseling for patients with normal bereavement reactions, and scared straight interventions. Only one participant explicitly mentioned Lilienfeld's (2007) paper, but some participants were aware of some of the potentially harmful treatments that are mentioned. One participant wrote about repressed memory techniques and critical incident stress debriefing:

Some examples that are highly relevant at the moment is to confess crimes that you did not commit, report false traumatic memories. Another treatment that comes to mind is etching in traumatic memories during debriefing. (Licenced psychologist, 39 years old)

#### Insufficient therapeutic alliance

Failing to establish a strong therapeutic alliance between the therapist and patient was declared a potential risk factor for treatment failure by several participants. It was for instance stated that the patient needs to understand the treatment rationale, the explicit goals of treatment, and what it means to achieve those goals in terms of time and commitment. One participant wrote:

Lacking a relationship, lacking an understanding of what the purpose of the treatment is, lacking the ability to meet the patient at the right level, or low motivation to change. All of these are factors that are important for both the therapist and the patient, that is, we as therapists need to adapt our behaviors to the patient's level in relation to several different parameters. (Licenced psychologist, 45 years old)

#### Failed ethical judgment and professional conduct

Other aspects of therapist behavior that were mentioned by many participants were failure to uphold the ethical standards and professional conduct that is expected by a practicing clinician. Particularly, violating or using your patient, as well as abusing your power as a therapist was considered to result in negative effects. One of the participants wrote about the obligation to adhere to ethical guidelines and to know your own limits as a therapist:

Unethical therapist behavior, disregarding professional and ethical guidelines. Or when the therapist takes on assignments within areas where the therapist in question lacks the necessary competence (which is also part of the professional responsibility to be able to determine). (Licenced psychologist, 55 years old)

Another participant wrote about meeting patients with a history of feeling misunderstood by their therapists and the negative consequences that may arise thereof:

I have seen quite a few patients, who have felt that they were not understood or validated, or even violated, and patients, who have been treated in such a way that they did not understand what the treatment was intended for. This in turn has led to more depression, anxiety, mistrust, etc.(Licenced psychologist, 45 years old)

#### Discontinuing treatment

Several participants discussed discontinued treatment as a potential risk factor for negative effects. Treatments can end prematurely by either the therapist or the patient. A number of participants described short-term negative effects as a potential cause for patients to leave treatment, but also unexpected attrition. A few participants also mentioned that some patients might not be able to pay for treatment. One participant wrote the following about making mistakes when having to end an ongoing treatment:

I myself have ended treatments in a clumsy way when I switched from one workplace to another, which led to disappointment which the patient had a hard time coping with. That is, thoughtlessness or carelessly administered interventions by therapists can create frustration, which overshadows the potential good that the therapist might have accomplished.—Licenced psychologist, 39 years old

Another participant wrote about how some patients leave treatment prematurely as a result of not being able to cope with the emotional stress and increase in anxiety that often accompany many interventions:

On a short term basis in the treatment of anxiety disorders: feelings of discomfort, increased levels of anxiety, and in certain cases the patient might not overcome that threshold and thus leave the treatment prematurely taking only negative experiences with her/him. (Licenced psychologist and licenced psychotherapist, 55 years old)

#### External factors

External factors were perceived by some participants as an important factor that might interfere with the quality and outcome of treatment, most notably the lack of funds to pay for the number of sessions required in order to benefit. Other types of external factors that might have a negative effect were the institution in which the therapist works, e.g., lack of support for the interventions that are deemed necessary, inadequate training, or financial restraints. One participant described experiencing financial pressures that affected the time allocated to psychological treatment in an outpatient care setting:

To rush through a treatment, to cut costs, it's like a surgeon sewing a patient back together with sloppy stitches. The surgery is done but the wound might not heal very beautifully. In my experience the outpatient care setting often struggles with demands of taking on high case-loads, which can result in shorter treatments, despite the evidence regarding how many sessions that are usually required to achieve a significant result. (Licenced psychologist, 52 years old)

#### Patients at higher risk of suffering from negative effects

In terms of patients that may have a higher risk of experiencing negative effects, none of the participants were able to distinguish any specific group or diagnosis. However, several participants mentioned patients with comorbid disorders as being more difficult to treat in general, and thus more likely to result in treatment failure or encountering negative effects. Some participants also believed that patients with a combination of personality disorders and anxiety disorders, or patients with low cognitive abilities and anxiety disorders, experience negative effects to a greater degree. One participant wrote about the delicate nature of treating individuals with personality disorders related to their lack of insights into their own responsibility in causing difficulties with others:

Individuals with a victim-mentality (everyone is mean to me and I have no impact on what happens in my life) often with personality disorders, are often discontent, they might even feel more violated and mistreated when you are not able to help them (since we often are not able to work directly with their home and social environment and only with the individual in question). They are often very upset if you suggest that they themselves might be part of their ongoing problem. (Licenced psychologist and licenced psychotherapist, 55 years old)

Another participant wrote about how you should address an anxiety disorder when there is an underlying personality disorder left untreated, which might make things worse:

If there is for example a personality disorder underneath and the treatment is aimed at panic disorder and/or depression, severe features of the personality disorder might be enhanced, in my experience. (Licenced psychologist, 52 years old)

Another participant wrote about the difficulties in providing the best intervention possible for individuals with varying capabilities and cognitive impairments, and how CBT might not always be the best choice:

The patients I see (an outpatient clinic for treating patients with psychosis) often have a history of failures or experiences of not achieving their treatment goals. To initiate psychological treatment when the patient might not have the capacity to carry out the tasks (I am specifically thinking of CBT because that is what I am trained to administer) might in the long run be less advantageous for the patient than for example counseling. The patient might lack the cognitive capacity, strength, and executive capability to carry out homework or other activities, which might cause the treatment to never really get off the ground or render any positive results. (Licenced psychologist, 26 years old)

### Criteria and methods for assessing negative effects

Participants were asked to propose specific criteria for assessing whether or not a patient had suffered from negative effects of psychological treatments. This was seemingly difficult to answer, and many of the participants discussed methods for assessing negative effects rather than defining specific criteria, e.g., therapist and patient assessment, qualitative data in the form of a patient's verbal account of the treatment and its perceived outcome, and the clinician's overall judgment of the patient's condition and development during treatment. However, a few participants mentioned a combination of measures concerning deterioration of illness, collection of qualitative data through clinical interviews, clinical judgment, as well as the systematic use of different outcome measures. One participant also mentioned the OQ-45 (Lambert, 2013) as a method for detecting negative effects during treatment. In addition, one participant highlighted the importance of considering the patient's own unique perspective:

I would first of all take into account what the individual tells me about her experiences of the treatment and how it has affected her/him. I'm not sure how to interpret ’criteria’. These would have to be based on the patient deteriorating, or on lack of treatment effects (that could have been obtained by another more effective treatment), and also on the notion that the treatment in question is faulty. (Licenced psychologist, 39 years old)

Furthermore, another participant wrote about taking the patient's perspective into account, as well as summarizing several different outcome measures in an overall judgment of the treatment outcome:

I usually evaluate treatments using measurements of symptoms, for example different scales and instruments, and often measurements of behavioral change, for example by registering target behaviors. Evaluation is also done verbally using questions about the patient's experiences of treatment and if any progress has been made. A clinical judgment about how the patient is doing after treatment has ended. I am lacking specific measurements of negative effects, but I think that if a significant deterioration has occurred, the methods mentioned above would make this easy to distinguish. (Licenced psychologist and licenced psychotherapist, 61 years old)

## Discussion

The purpose of the current study was to investigate how clinicians perceive and experience negative effects in their clinical practice, as well as to examine their knowledge of the available research findings regarding negative effects in psychological treatments. The results reveal that a majority of the participants agreed that negative effects of psychological treatments exist and pose a problem, and many were also able to recount incidences where their patients had either deteriorated or encountered adverse events. In addition, the results suggest that there is a lack of consensus regarding how and by what criteria negative effects should be determined. Most of the participants responded by discussing measurement issues rather than to provide a definition of negative effects, e.g., to use clinical judgement or rely on validated outcome measures, as well as presenting various types of negative effects, e.g., short-term negative effects, no treatment effect, and impact on other life domains. This finding is, however, in line with prior research where the conceptualization of negative effects ranges from deterioration to misuse of psychological treatments, novel symptoms, social stigmatization, interpersonal difficulties, and lowered self-esteem (Dimidjian & Hollon, 2010; Lilienfeld, 2007; Boisvert & Faust, 2003), while the criteria for assessing negative effects are still unclear, making it complicated for clinicians to distinguish negative effects in clinical practice (Peterson et al., 2013). Hence, a more uniform classification for defining negative effects could become valuable in order to raise the awareness among clinicians of what negative effects that can occur. Likewise, the different suggestions on how to monitor negative effects that recently have been proposed may facilitate the investigation of deterioration and adverse events by providing useful measures and questionnaires to clinicians and researchers (Linden, 2013; Nestoriuc & Rief, 2012; Parker et al., 2013).

The results of the current study also showed that the participants were able to discuss a number of factors that are presumed to be related with the occurrence of negative effects in psychological treatments, e.g., the therapeutic relationship, the importance of a mutual agreement, certain patient groups, as well as adequate competence and proper application of interventions. Although the causality of psychological treatments and negative effects is difficult to examine, similar factors are discussed by Castonguay et al. (2010), suggesting that the clinician's behavior, rigid application of a therapeutic model, and specific patient characteristics might result in a greater incidence of negative effects. Understanding what factors could be associated with deterioration and adverse events is deemed important, and should help clinicians prevent and manage negative effects in their clinical practice. However, this warrants an increased acknowledgement of negative effects during basic clinical training, which the current study indicates might not be the case. Only a small portion of the participants responded to having received information of negative effects through literature and research, and when considering the range in age and years of clinical practice among the participants, this is assumed to be lacking still. Castonguay et al. (2010) have presented a number of guidelines in order to introduce the idea of negative effects to clinicians in Spe, e.g., clinical supervision focusing on how to determine lack of improvement, systematic evaluation of treatment progress, as well as knowledge of the therapeutic variables and patient characteristics that can contribute to deterioration and adverse events, and may help to improve the awareness of negative effects in basic clinical training.

The current study has several methodological limitations that need to be considered when reviewing the results. First, of the approximately 1400 potential participants who received an emailed invitation to participate, only 74 completed the survey, generating a response rate of no more than 5%. The survey might therefore have been affected by response bias, which can in turn impact the generalizability of the results. However, this is in line with the response rate of similar investigations, e.g., 12% for Hatfield et al., (2010), even though Boisvert and Faust (2006) did manage to increase their response rate to 36% by using additional reminders that were sent out by post cards. The use of email in the current study could be a possible explanation for the small number of participants completing the survey, but it is also unclear how many of the 1400 potential participants actively receive email from their respective affiliation. Furthermore, the deadline for the survey was only two weeks, and no financial compensation was offered, which might have influenced the motivation to participate. Second, the use of a survey and not an interview might have restricted the type of responses that the current study was able to collect. The participants were for instance unable to elaborate their responses after submission, and no follow-up questions were feasible, ruling out the possibility to probe for additional information. Third, the thematic analysis was conducted entirely by the first author of the current study, and no reliability estimate such as inter-rater reliability by assessing Cohen's kappa or percentage agreement was used. This may have affected the coding of the results and increased the risk of performing human errors. However, methodological issues and difficulties related to the analytic procedure were discussed together with the co-authors, and the themes and formulations of themes were also reviewed by the second author. In addition, by providing facts on how the participants were recruited, their occupation and theoretical orientation, and the process of analyzing the data, greater transparency has been achieved in the current study, thus enhancing the credibility and transferability of the results (Sandelowski, 2000). Recommendations for future research is to use an interview in order to further explore the perception and experiences of negative effects among clinicians, as well as to investigate what measures and criteria that can be used to identify patients at risk of deterioration or adverse events.

## Appendix

1. Age
2. Gender
3. I practice psychological treatment as part of... (what occupation)?
4. Please describe your basic clinical training
5. My main therapeutic orientation, that is, the theoretical perspective I rely on when practicing psychological treatment, is...
6. How many years have you been practicing psychological treatment?
7. Is there a problem of negative effects, i.e., can we legitimately speak of a patient getting worse or experiencing adverse events as a result of psychological treatment?
8. If so, what would constitute a negative effect in psychological treatment?
9. By what criteria would one judge a patient as having experienced negative effects as a result of psychological treatment?
10. While any therapy outcome is obviously a function of many factors, which factors would you associate with, or consider responsible for, a negative effect?
11. In your opinion, is there a particular patient group that might experience negative effects of psychological treatment to a greater degree?
12. Did you during your basic clinical training receive information about, or come in contact with any literature or research that concerns negative effects of psychological treatments?
13. Do you have any clinical experience of negative effects of psychological treatments?
14. Is there anything else you would like to add?
